# Immunonutrition: Another Player on the MASLD Field

**DOI:** 10.3390/ijms26188928

**Published:** 2025-09-13

**Authors:** Iván López-Méndez, Misael Uribe, Eva Juárez-Hernández

**Affiliations:** 1 Hepatology and Transplants Unit, Medica Sur Clinic & Foundation, Mexico City 14050, Mexico; 2 Gastroenterology and Obesity Unit, Medica Sur Clinic & Foundation, Mexico City 14050, Mexico; muribe@medicasur.org.mx; 3 Translational Research Unit, Medica Sur Clinic & Foundation, Mexico City 14050, Mexico

**Keywords:** inflammation, oxidative stress, liver steatosis, nutrition

## Abstract

Immunonutrition is a nutritional strategy where the bioactive properties of nutrients from the diet are used to modulate metabolic pathways, inflammation signals, and oxidative stress regulators. Metabolic dysfunction-associated steatotic liver disease (MASLD) is a chronic degenerative disease with increasing prevalence over the past decade. In MASLD, where inflammation and oxidative stress play key roles in the progression of liver disease, immunonutrition becomes even more important. The impact of different dietary patterns has been studied in the MASLD context; however, current guidelines emphasize the Mediterranean Diet, which, in terms of included food groups, provides a high level of immunonutrients. Nonetheless, adherence, monitoring, and implementation based on geographic areas, availability, and economic factors make this type of diet ultimately less accessible. The main benefit of the diet pattern is in maintaining the positive effects of new pharmacological treatments, alongside physical activity, which are crucial to prevent recurrence. A diet strategy designed for MASLD needs to be adapted to local food availability and should promote the inclusion of immunonutrients. In the treatment of MASLD, dietary recommendations should be individualized based on the sociodemographic, clinical, and nutritional characteristics of the patients.

## 1. Introduction

Immunonutrition is a nutritional strategy in which the bioactive properties of nutrients obtained from the diet are used for the prevention and treatment of conditions characterized by infection, inflammation, or tissue damage [1]. According to this concept, immunonutrients are defined as those nutrients that have been shown to directly or indirectly affect the immune response, synergize cellular immunity, and interact with the metabolism of inflammatory cells through their antioxidant properties, antibody production, immune cell activation, and regulation of inflammatory processes [2].

Chronic degenerative diseases, primarily associated with metabolic disorders, are currently the most prevalent and the leading causes of mortality worldwide. In their pathophysiology, all of them involve the activation of inflammatory processes, oxidative stress, and tissue damage [3], which is why immunonutrition has been proposed as an additional therapeutic tool. Metabolic dysfunction-associated steatotic liver disease (MASLD) is a chronic degenerative disease defined as the accumulation of more than 5% fat in the liver parenchyma, in combination with the alteration of markers associated with metabolic syndrome [4]. MASLD prevalence has increased by 50% in the last decade, with an estimated worldwide prevalence of 38%, being higher in Latin America (44%), which places this disease as the first indication for liver transplant. Current evidence shows that patients with MASLD can progress to liver cirrhosis within 10 to 15 years; this progression is faster (7 to 10 years) in the presence of metabolic dysfunction-associated steatohepatitis (MASH) [5]. In the progression of MASLD to MASH and liver fibrosis, inflammation, oxidative stress, and tissue damage play an important role in the development of cirrhosis and hepatocellular carcinoma [6].

The synthesis and metabolism of nutrients, mainly in the liver, are an important factor in the immune system’s response capacity. Therefore, the presence of liver damage may represent an immunological limitation against inflammatory mechanisms [7]. It has been described that the regulation of immunonutrients through diet or supplementation could modulate liver inflammation, regulating the production of proinflammatory cytokines and nuclear factor kappa B (NF-κB), both of which play an important role in the main pathological pathways of MASLD, such as insulin resistance (IR) and cardiometabolic disorders [8]. Even when immunonutrients supplementation could be an additional option, these could be obtained through a balanced diet (Table 1).

Although several pharmacological treatments have demonstrated efficacy in MASLD, lifestyle modification remains the cornerstone of treatment; in this setting, the addition of immunonutrition could be beneficial in reducing the inflammatory activity of the disease [1]. This review aims to describe the evidence generated to date on the effect of immunonutrition in MASLD.

## 2. Background

The benefits of immunonutrition as an additional therapeutic strategy have been demonstrated in various clinical settings, including oncological and surgical processes, trauma, and critical illness, with an impact on important outcomes such as reduced hospital stays, days on mechanical ventilation, reduced inflammation, a lower risk of infection, and improved cicatrization [9,10,11]. Despite these benefits, existing evidence on the effect of immunonutrition in liver diseases is limited. The main findings were observed in patients with cirrhosis, in whom supplementation with glutamine, L-arginine, and omega-3 (ω-3) and omega-6 showed improvement in the total score of the Subjective Global Assessment (16.3 ± 3.8 vs. 11.2 ± 2.2, *p* < 0.001) in patients on the liver transplant waiting list; however, no significant differences were observed regarding anthropometric parameters [12].

Currently, MASLD is the most common cause of liver disease [13] with an estimated prevalence of 25–40% [5]. MASLD is a multicausal entity closely related to overweight, obesity, and other metabolic disorders. Individually, immunonutrition has been shown to have beneficial effects in terms of weight loss and improvement in serum glucose, lipids, and blood pressure levels [14,15].

MASLD is a metabolic disease with a complex physiopathology that begins with cytoplasmic hepatic fat accumulation (triglycerides) and IR, leading to lipotoxicity and the stimulation of de novo fatty acid synthesis by excess carbohydrates. Subsequently, endoplasmic reticulum stress, the production of reactive oxygen species (ROS), oxidative stress, and organelle dysfunction cause liver injury, which progresses to steatohepatitis. In the presence of overweight or obesity, the release of inflammatory mediators contributes to hepatocyte damage, leading to ballooning, activation of hepatic stellate cells (HSC) (also associated with IR), and sinusoidal collagen deposition, which results in fibrosis—the next stage in the progression of MASLD [16].

In MASLD development pathways, the dysregulation of AMP-activated protein kinase (AMPK), mechanistic target of rapamycin (mTOR), and Sirtuin 1 (SIRT1) plays an important role due to their functions in glucose and lipid metabolism, lipid and protein synthesis, inflammation, and autophagy. Hence, some therapeutic options for MASLD involve targeting the signaling of these proteins.

AMPK phosphorylates sterol regulatory element-binding protein 1c (SREBP1c), which is associated with the regulation of lipid, insulin, and glucose metabolism. This phosphorylation has a direct effect on reducing hepatic steatosis and diet-induced IR, as well as decreasing the expression of fatty acid synthase and inhibiting lipid synthesis. On the other hand, AMPK is associated with negative feedback to mTORC1, which also activates SREBP with effects on hepatic lipogenesis. The improvement in AMPK signaling has been associated with the suppression of proinflammatory cytokines, specifically TNF-α, IL-1β, and IL-6, through the activation of SIRT1, a protein that regulates inflammation and DNA repair. Proper AMPK signaling is crucial for suppressing inflammation, inhibiting hepatic stellate cell activation, and improving liver injury by regulating mitochondrial function and cell death pathways [17,18].

Immunonutrition principles indicate that it could have a beneficial effect on the different pathophysiological characteristics of MASLD: (1) oxidative stress generated by a sedentary lifestyle and deficient diets in quality and quantity, (2) the inflammatory process secondary to damage induced in the hepatocyte, and (3) tissue damage produced by the generation of free radicals and the production of proinflammatory cytokines that leads to fibrosis and cirrhosis development [19].

Taking these pathophysiological mechanisms into account, immunonutrients have specific effects on the activation or inhibition of the immune system through the acceleration of T cell-mediated immunity, modifying the body’s response to inflammation by mediating the production of proinflammatory cytokines, maintaining the intestinal barrier function through the activation of gut-associated lymphoid tissue, and improving the antioxidant status, with antioxidant vitamins, specific amino acids, polyphenols, and fatty acids being the main immunonutrients involved [20,21].

As mentioned, the pathophysiology of MASLD involves various mechanisms, which make the search and development of therapeutic options difficult in a context with multiple altered and interconnected pathways. While the efficacy of therapies for MASLD is based on histopathological outcomes related to the resolution of progression to steatohepatitis and fibrosis, the clinical and epidemiological context of this liver disease requires that other factors be considered as therapeutic targets. The effect of nutritional strategies is mostly evaluated through modifications in serum lipid levels, protein expression, and changes in liver function tests, which are useful as biomarkers of liver damage with good utility in clinical practice to evaluate the severity, progression, and response to treatment in MASLD [22].

Nowadays, several immunonutrients have been shown to have beneficial effects in various clinical settings; however, in other clinical scenarios, such as MASLD, the evidence regarding the beneficial effects of immunonutrients decreases. In this review, we present evidence of those immunonutrients that have been associated with beneficial effects in different outcomes of MASLD.

**Figure 1:**
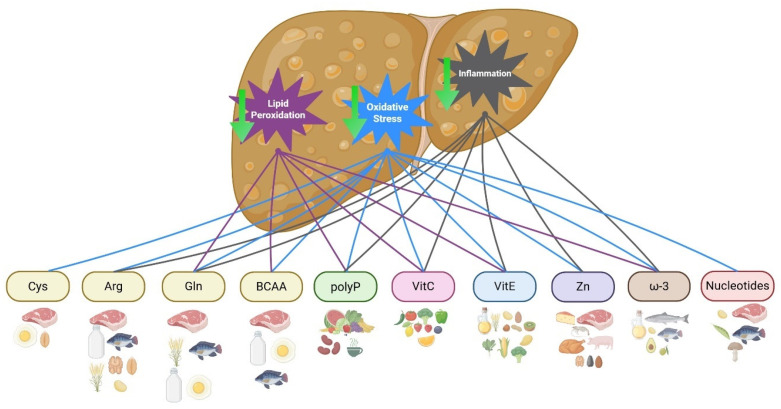
Effects of immunonutrients in MASLD pathways. The figure shows the dietary source of each immunonutrient and its effect on decreasing lipid peroxidation, oxidative stress, or inflammation. All immunonutrients are associated with the decrease in oxidative stress; meanwhile, Arg, Gln, polyp, vitamins, inorganic nutrients, and ω-3 are related to decreasing inflammation markers. Gln, BCAA, polyP, vitamins, inorganic nutrients, and ω-3 have also been associated with a decrease in lipid peroxidation. Cys, Cysteine; Arg, Arginine; Gln, Glutamine; polyP, Polyphenols; VitC, Vitamin C; VitE, Vitamin E; Zn, Zinc; ω-3, omega-3 fatty acid. Created in BioRender. Juarez-Hernández, E. (2025).

In this metabolic–hepatic disease, where inflammation and oxidative stress are determining factors for the progression of liver disease, the functions of immunonutrition with respect to inflammation and its antioxidant effect become more important; immunonutrients such as ω-3 fatty acids and vitamins E, C, and D are involved in decreasing the production of proinflammatory cytokines, while amino acids and polyphenols have been associated with antioxidant properties; on the other hand, zinc (Zn) is an immunonutrient with the ability to modulate different pathological pathways in the development or progression of MASLD [23] (Figure 1).

## 3. Immunonutrients in MASLD

### 3.1. Amino Acids

#### 3.1.1. Cysteine

Cysteine is a conditionally essential amino acid synthesized from the metabolism of methionine through transsulfurization, which promotes the production of glutathione. Cysteine is used for protein synthesis and can be converted into taurine, glutathione, and hydrogen sulfide. In addition to being synthesized from methionine, cysteine can be obtained in the diet through the intake of meat, eggs, and whole grains [24].

Cysteine interacts with glycols to form glycoproteins, such as secretory acidic protein rich in cysteine (SPARC), an extracellular matrix glycoprotein expressed in adipose, liver, muscle, and pancreatic tissue. Studies in murine models have shown that SPARC contributes to adipose tissue formation and cell differentiation, in addition to modulating the function of cell adhesion molecules at the endothelial level, remodeling the extracellular matrix [25]. Furthermore, cysteine enables immune cells and hepatocytes to combat oxidative stress caused by lipid accumulation, a characteristic of steatotic liver disease (SLD) caused by lipid extravasation from the vascular endothelium [26].

Regarding the role of cysteine in the liver, SPARC is expressed and secreted by HSC and hepatic endothelial cells in response to tissue damage. Upon activation, HSC differentiate into myofibroblast-like cells and express collagen 1-alpha production [27,28].

This process is key in the development of liver fibrosis due to MASLD. Furthermore, it has been shown to contribute to inflammation, which, in conjunction with lipid accumulation, induces tissue oxidative stress that leads to lipid peroxidation. Several studies, mainly in murine models, have demonstrated the role of SPARCs in the formation of adipose tissue in diabetes and SLD. Therefore, they have been proposed as a therapeutic target and a biomarker in the progression of diseases related to the accumulation of adipose tissue, with alterations in muscle metabolism [29].

Cysteine inhibition appears to be related to glucose disturbances and the development of MASLD; Tran et al. [30] evaluated the effect of SerpinA3N, a cysteine inhibitor protein, in a mouse model of SLD induced by a methionine- and choline-deficient diet. SerpinA3N knockout mice showed a significant reduction in adipose tissue, with improved glucose tolerance and insulin sensitivity. Therefore, it is proposed that this protein could be involved in glucose homeostasis and, therefore, in MASLD development.

In its pharmacological form, cysteine is presented as N-acetylcysteine (NAC), which is approved for the treatment of respiratory conditions involving mucous secretions, prevention of nephropathy, and acute liver failure caused by toxic doses of acetaminophen, due to its hepatoprotective effect [31]. Specifically in MASLD, the antioxidant effect of NAC has been studied as an adjuvant treatment option. In a murine and cellular model of MASLD, Hang et al. [32] reported that the administration of NAC significantly reduced serum and histological triglyceride levels (*p* < 0.05) and preserved mitochondrial function, preventing the production of ROS. Despite these results, a recent meta-analysis evaluating the effect of NAC on liver function tests showed that NAC administration did not improve serum levels of (Aspartate aminotransferase) AST (SMD −0.03 (−0.53, 0.47), *p* = 0.917; ALT (Alanine aminotransferase) (SMD −0.22 (−0.63, 0.19) 0.288) and (Alkaline phosphatase) ALP (SMD −0.22 (−0.67, 0.23) 0.343, *p* = 0.33). It is worth noting that these results are from 640 patients in eight studies, only two of which included patients with liver disease [33].

The antioxidant effect of cysteine in patients with MASLD requires further evidence to be recommended as an additional treatment option.

#### 3.1.2. Arginine

Arginine is an amino acid synthesized by the urea cycle in the liver, which functions in protein synthesis and the generation of polyamines and creatine to maintain cellular homeostasis. Arginine stimulates nitric oxide synthesis and growth hormone production, with an anabolic effect [34]. Arginine is obtained in the diet from the consumption of meat, fish, nuts, wheat germ, whole grains, soy, and dairy products [35,36].

Through cellular redox regulation, arginine prevents the oxidative stress that is characteristic of MASLD development [37]. Arginine has an antioxidant effect by reducing oxidative stress through restoring glutathione and decreasing TNF-α levels, as well as decreasing inflammation by reducing neutrophil infiltration [38].

Since its main sources are animal products, a deficiency in protein intake, and therefore arginine deficiency, has been associated with triglyceride accumulation in the liver. In a mouse model, Otani et al. [39] evaluated this accumulation in three groups: a control diet with normal protein distribution (15%), a diet low in total amino acids, and a diet low in arginine. They observed that arginine and total amino acid deficiency caused significant triglyceride accumulation. Two mechanisms were proposed: an increase in signaling proteins that promote lipogenesis due to protein deficiency, and very low-density lipoprotein secretion due to a reduction in ApoA4 expression due to arginine deficiency. It was concluded that low-protein and low-arginine diets could contribute to the development of MASLD associated with increased lipid accumulation in the liver.

In a study with a murine model, in which steatohepatitis was developed by intravenous administration of 20% lipids for 3 weeks and were pre- and post-treated with arginine injection for 2 weeks, it was shown that the administration of arginine significantly decreased the levels of lipid peroxidation and the enzyme CYP2EI (0.32 ± 0.01 vs. 0.3 ± 0.02 IU/mg), markers of oxidative stress, indicating that arginine reduces it. Arginine supplementation also showed a significant increase in nitric oxide levels (1.649 ± 0.047 vs. 1.957 ± 0.073 μmol/g) and in endothelial nitric oxide activity at the hepatic level (0.05 ± 0.002 vs. 0.056 ± 0.002 IU/mg), as well as a significant decrease in TNF-α levels, both at the blood level (4.5 ± 0.08 vs. 4.12 ± 0.13) and at the hepatic level (13.1 ± 0.6 vs. 11.9 ± 0.5), decreasing inflammation [40]. This antioxidant effect can be attributed to the alpha-amino group, necessary for nitric oxide production; hence, treatment with arginine could restore antioxidant enzyme levels, such as glutathione, at the liver level.

#### 3.1.3. Glutamine

Glutamine is the most abundant amino acid in the human body, accounting for approximately 60% of the total protein. Therefore, it plays an important role in maintaining and promoting various physiological functions such as protein synthesis, immune system activation, and mucosal protection. Therefore, its concentration has been associated with the pathophysiology of various chronic diseases [41]. Although glutamine is not an essential amino acid, it can be obtained from the diet through the consumption of meat, eggs, fish, dairy products, wheat, tofu, and some fermented products [42,43]. Glutamine is crucial for energy metabolism and hepatocyte proliferation; it is also an important precursor for gluconeogenesis. Metabolic stress, characteristic of SLD, attenuates endogenous production [44].

Due to glutamine’s role in maintaining mitochondrial metabolism and cell proliferation, alterations in its metabolism, produced by the progression of inflammatory diseases and metabolic stress, such as those occurring in chronic liver disease, have been associated with changes in cell cycles. Specifically in the liver, increased glutaminolysis, with the consequent conversion of glutamine to glutaminase and glutathione, has been associated with the proliferation and activation of HSCs. Therefore, glutamine metabolism regulation has been proposed as a therapeutic target for the presence and progression of liver fibrosis. However, these proposals arise from studies in cell lines and mouse models of liver fibrosis. More evidence is needed to determine the effect and mechanisms of glutamine modulation across the spectrum of liver disease [43,45,46,47].

Glutamine has been described as a protective factor against liver diseases, such as alcoholic liver disease and MASLD. This is due to its protective effect against oxidative stress and the reduction in proinflammatory cytokines production. Glutamine helps maintain adequate glutathione levels, which allows hepatocytes to combat oxidative stress and eliminate toxins [48].

In a study with rats with High-Fat Diet (HFD)-induced MASLD, glutamine (1 g/kg) was administered, oxidative stress levels were measured over different periods, and the regulatory effect of glutamine on oxidative stress due to MASLD was analyzed. It was found that high levels of oxidative stress and inflammation were reduced with dietary glutamine supplementation, thanks to the inhibition it causes in the proinflammatory gene transcription pathway NF-Κb [49].

MASLD patients present a persistent chronic inflammatory process that generates activation of the innate immune system, releasing inflammatory mediators such as TNF-α and increasing mRNA expression [50]. The transcriptional process associated with the production of proinflammatory cytokines is primarily mediated by the NF-κB pathway. In the study by Lin et al. [49] glutamine supplementation in rats showed significant changes in TNF-α (275.93 ± 34 vs. 343.83 ± 20.61 ng/L) and malondialdehyde (0.90 ± 0.6 vs. 1.11 ± 0.10 nmol/mg), as well as histological changes in steatosis and NF-κB degree, in addition to the expression of proinflammatory cytokines.

Despite these positive results, it is important to consider that they are based on murine models, so it is necessary to evaluate the effect of glutamine supplementation in patients with MASLD.

#### 3.1.4. Branched-Chain Amino Acids

Branched-chain amino acids (BCAAs) are leucine, isoleucine, and valine. They are essential amino acids, so they must be obtained from the diet. The primary sources of these amino acids are animal products, primarily including meat, fish, dairy, and eggs. BCAAs are necessary for protein synthesis and maintaining energy balance, as well as various metabolic processes, including the regulation of lipids and glucose [51,52].

Specifically, leucine activates the mTORC1 complex, which regulates cell growth and metabolism [53] with an effect on protein metabolism and the promotion of protein synthesis [54]. At the hepatic level, different metabolic pathways involving BCAAs have been described, mainly those associated with glucose regulation and insulin signaling [55].

In the metabolism of BCAAs, there are different interactions with the oxidation of fatty acids and de novo lipogenesis at the hepatic level [56]. The effect of BCAAs on MASLD is not fully described; however, one of the involved pathways is the increase in portal production of acetic acid due to the rise in bacteria of the genus Ruminococcus, specifically R. flavenfaciens, which reduces liver fat accumulation in murine models [53].

In a study with steatohepatitis mice induced by a choline-deficient HFD, the effect of BCAAs supplementation was evaluated after 8 weeks. Honda et al. [54] observed a significant reduction in serum ALT levels [263.2 ± 30.5 U/L vs. 252.5 ± 19.5 U/L, *p* < 0.0001] and glucose (62.2 ± 6.1 ng/mL vs. 58.3 ± 2.8 ng/mL, *p* < 0.01), as well as hepatic triglyceride levels. The expression of fatty acid synthase genes also decreased following supplementation.

Regarding the antioxidant functions of BCAAs, in a murine model, Surugihalli et al. [56] evaluated the effect of BCAA supplementation in combination with HFD, observing that this supplementation was associated with a higher nicotinamide adenine dinucleotide (NADH):NAD+ ratio and activation of hepatic AMPK, leading to a change in redox status that increased fatty acid oxidation.

Although the benefit of BCAA supplementation has sufficient evidence in end-stage liver disease, despite its relationship with protein synthesis and lipid metabolism, in the context of MASLD, more evidence is still needed to support BCAA supplementation as an adjuvant in the treatment.

### 3.2. Polyphenols

Polyphenols are non-energetic organic compounds, characteristic of plants, with a molecular structure defined by the presence of phenolic units or rings. Polyphenols are grouped into two main classes: (1) flavonoids, including isoflavones, quercetins, cyanidins, and catechins, and (2) phenolic acids, which include caffeic acid and ferulic acid [57,58]. Polyphenols have been shown to play an important role in scavenging ROS and improving lipid profiles, IR, and systemic inflammation [59].

The antioxidant effect of polyphenols has been demonstrated in chronic degenerative diseases such as diabetes and obesity. Flavonoids such as anthocyanins, flavonols, and flavanols act by increasing aerobic lipid metabolism and mitochondrial bioenergetics [60]. Flavonoids have been attributed antioxidant, anti-obesogenic, and chemoprotective properties by scavenging free radicals and activating molecular effectors implicated in various diseases. Most dietary flavonoids are glycosylated, which limits their absorption in the small intestine and, therefore, their systemic distribution. As a result, they become a substrate for intestinal microbial catabolism upon entering the colon [61].

One of the polyphenols with the most significant evidence of beneficial effects on inflammatory diseases in the last decade is curcumin. It is a polyphenolic compound found in the plant Curcuma longa, known as turmeric. It has been studied for its anti-inflammatory and antioxidant properties and for its potential anticancer effects [62,63,64]. Despite its therapeutic properties, it has low bioavailability in the body due to its low solubility in water and its rapid metabolism and excretion in the body; however, different types of curcumin improve bioavailability, such as phytosomal curcumin, piperine and nano-micellar [65,66].

The main dietary source of polyphenols is the consumption of foods characteristic of the Mediterranean diet (MedDiet), such as plants, fruits, chocolate, legumes, turmeric, and beverages such as green tea and wine [58]. Polyphenol consumption or supplementation has demonstrated beneficial effects on cardiovascular risk outcomes; however, catechins and turmeric are the polyphenols with the greatest evidence of beneficial effects on SLD. In different clinical and preclinical studies, it has been observed that polyphenols decrease proinflammatory cytokines both in serum and at the histological level, which contributes to a decrease in fatty liver, in addition to being able to improve the regulation of adipocytes and prevent MASLD [67].

Catechins, polyphenols characteristic of green tea, have been shown to have therapeutic effects in patients with diabetes mellitus and metabolic syndrome through the regulation of glucose and lipid metabolism, improving glucose synthesis and inhibiting lipogenesis in hepatocytes. In addition, they mobilize and activate endogenous antioxidant mechanisms, improving the activity of enzymes such as superoxide dismutase, decreasing lipid peroxide levels in the plasma and, therefore, detoxifying the liver during metabolism [68].

Epigallocatechin-3-gallate (EGCG) is the most active compound in green tea, so its therapeutic effect has been studied in different MASLD outcomes. In a liver cell line, Wu et al. [69] observed that EGCG reduces mitochondria-dependent apoptosis, increasing autophagy through mitogen-activated protein kinase, which is mediated by ROS. These results were confirmed in the same study, in a murine model of MASLD induced by HFD. In a murine model of MASLD induced by fructose and HFD, a low and high dose of EGCG were administered for 8 weeks, observing a 47% reduction in hepatic triglycerides (*p* < 0.01) and a 38% reduction in serum cholesterol (*p* < 0.05). At the histological level, treatment with EGCG showed a 50% reduction in the steatosis score and a 57% reduction in the steatohepatitis score (*p* < 0.0001), with improvement in inflammation degree [70].

Curcumin has been a substance of interest in various MASLD studies since its antioxidant and anti-inflammatory properties have proposed it as a possible therapeutic strategy [71]. In a murine model of HFD-induced MASLD, the effect of curcumin supplementation was evaluated, observing improvements in hepatic endothelial function, reducing hepatic lipid accumulation and inflammation. This suggests that curcumin exerts an inhibitory mechanism on the NF-κB and PI3K/Akt/HIF-1α pathways. Similarly, it could reduce lipid accumulation in the liver by inhibiting citrate transport and metabolism, which is crucial for de novo lipogenesis [72].

A randomized clinical trial of 80 patients with MASLD reported that supplementation with 500 mg of curcumin for 24 weeks significantly reduced the degree of hepatic steatosis, assessed by transient elastography, compared to the placebo group and other parameters such as body weight by 2.6 kg (95% CI: −4.4 to −0.8 kg; *p* < 0.001), body mass index by 1.0 kg/m^2^ (95% CI: −2.0 to −0.1 kg/m^2^; *p* = 0.032), free fatty acids by 0.12 mmol/L (95% CI: −20 to −0.04 mmol/L; *p* = 0.004), triglycerides by 0.29 mmol/L (95% CI: −0.41 to −0.14 mmol/L; *p* < 0.001), fasting glucose of 0.06 mmol/L (95% CI: −0.12 to −0.01 mmol/L; *p* = 0.038), hemoglobin A1c of 0.06% (95% CI: 0.33 to −0.01%; *p* = 0.019), and insulin of 4.94 μU/L (CI 95%: −9.73 to −0.15 μU/L; *p* = 0.043) [73].

A meta-analysis of 16 randomized clinical trials evaluating different curcumin formulations showed that curcumin improved serum liver enzyme levels (AST) by an average of −3.90 U/L (95% CI: −5.97, −1.82) and ALT by an average of −5.61 U/L (95% CI: −9.37, −1.85), and reduced fasting glucose, total cholesterol, and body mass index. Regarding histological outcomes, curcumin supplementation showed resolution of steatosis (RR 3.53 (95% CI: 2.01, 6.22)) as well as a reduction in steatosis severity (RR 3.41 (95% CI: 1.36, 8.56)). While these results support the beneficial effect of curcumin supplementation in MASLD, it is important to note that this meta-analysis focuses on a single geographic region (Iran and Thailand) [66].

Based on the results of preclinical and clinical studies, polyphenols may have therapeutic effects in MASLD, with the advantage of their availability in the diet; however, the most effective polyphenols in preventing MASLD, as well as the specific molecular mechanisms through which they act, are currently unknown.

### 3.3. Vitamins

#### 3.3.1. Vitamin C

Vitamin C is a fat-soluble vitamin known for its potential antioxidant effects, as well as for its role as a cofactor that scavenges free radicals and protects the body from tissue damage and oxidative stress [74]. The main sources of vitamin C are fruits and vegetables, primarily citrus fruits, berries, tomatoes, peppers, and leafy greens such as broccoli, kale, and parsley [75,76].

In MASLD, evidence on the role of vitamin C is limited, with most studies in murine models; however, it has been suggested that, due to its antioxidant effect and its role in enzymatic reactions, vitamin C is involved in lipid regulation in the liver [77].

In mice induced with MASLD using a high-fructose diet and HFD and administered a megadose of vitamin C for 11 weeks (1.5 g/L), disease progression, inflammation, and triglyceride levels were measured. Histological changes, such as less lobular inflammation and fat accumulation, as well as a reduction in the accumulation of triglycerides and free fatty acids in the liver, were observed. Neutrophil infiltration and the expression of proinflammatory genes (IL-1β, IL-6) were also decreased, thereby reducing inflammation. It was concluded that vitamin C supplementation of 1000 mg/day may be a treatment option worth evaluating in clinical trials to prevent disease progression [78].

Currently, studies confirming the effect of vitamin C in human patients with MASLD are limited; however, a randomized, double-blind clinical trial conducted in China evaluated the effect of vitamin C supplementation for 12 weeks in patients diagnosed with MASLD divided into three groups: low dose (250 mg), medium dose (1000 mg), and high dose (2000 mg). Medium supplementation was shown to decrease ALT levels [mean, −8.00 (−18.00, −1.75) vs. high, −3.50 (−13.75, 4.25), *p* = 0.05; medium vs. low, −3.00 (−9.00, 5.50), *p* = 0.031] and AST [medium, −5.00 (−10.25, −1.75) vs. high, −2.50 (−7.75, 0.00), *p* = 0.02]. Regarding the effects on glucose alterations, the medium dose also showed significant differences in HOMA-IR (before 4.61 ± 3.51 vs. after 2.62 ± 2.41, *p* < 0.001) [79].

Although there is evidence confirming the anti-inflammatory effect and improvement of metabolic markers of vitamin C supplementation, it is still insufficient to make a therapeutic recommendation for patients with MASLD.

#### 3.3.2. Vitamin E

Vitamin E is a fat-soluble vitamin located primarily in the cell membrane, where it exerts its primary antioxidant effect. It is the first defense against lipid peroxidation, protecting membranes from the action of free radicals, which is why it is considered the ultimate antioxidant. Vitamin E groups together various compounds, including tocopherols and tocotrienols [80]. It is obtained from the diet and its main sources are vegetable oils (wheat germ, sunflower, corn, soybean) and nuts, such as almonds, peanuts, and hazelnuts; however, it can also be found in green leafy vegetables, such as broccoli and spinach, in fruits such as kiwi and mango, and in fortified cereals [81].

In MASLD, vitamin E improves lipotoxicity by suppressing lipid accumulation and peroxidation, with effects on inflammation and IR by inhibiting the activation of M1-like macrophages/KCs. Vitamin E has also been shown to decrease HSC activation [82]. Levels of antioxidants such as α-tocopherol are decreased in patients with MASLD; when depleted, vitamin E’s regulatory capacity to control oxidative stress in the liver is diminished [83].

Among the immunonutrients, vitamin E has the most significant evidence of a beneficial effect in MASLD treatment. The PIVENS study [84] demonstrated that, compared with placebo, vitamin E not only had an effect on reducing liver enzymes (ALT: −20.1 vs. −37.0, *p* = 0.001; AST −2.8 vs. −21.3, *p* < 0.001, γ-glutamyl transferase −4.0 vs. −14.0, *p* = 0.003), but also produced significant improvements in MASH histological markers such as lobular inflammation (31 vs. 54%, *p* = 0.02), hepatocellular ballooning (29 vs. 50%, *p* = 0.01), and the SLD activity score (mean change −0.5 vs. −1.9, *p* < 0.001).

Due to this and other important results, current clinical practice guidelines for MASLD recommend the use of vitamin E at a dose of 800 IU/day as a treatment option for patients with MASLD without diabetes and without risk factors for prostate cancer [85].

The effect of vitamin E in combination with phenolic compounds has recently been studied; the study by Lahmi et al. [86] demonstrated in a murine model, in which MASLD was induced by a high fat emulsion and were supplemented for 28 days, that AST and ALT levels decreased in the groups supplemented with 200 mg/kg/day of vitamin E and in the group supplemented with vitamin E and thymol (50 mg/kg/day), a phenol compound found in the essential oils of thyme and oregano. Similarly, these groups demonstrated a significant decrease in cytoarchitectural changes in hepatocytes compared to the control group, concluding that supplementation with vitamin E and thymol could positively contribute to mitigating the damage caused by MASLD combating oxidative stress.

### 3.4. Trace Elements

#### Zinc

Zn is the second most concentrated trace element in the body and is involved in multiple signaling, regulatory, structural, and catalytic functions. It has an anti-inflammatory role in energy and antioxidant metabolism. It is a cofactor for more than 300 enzymes, and more than 3000 proteins are dependent on Zn concentrations, thus playing an essential role in cellular regulation and metabolism [87]. The primary dietary sources of Zn are seafood, beef, pork, chicken, cheese, and seeds such as almonds, sunflowers, and cashews [88].

There is a close relationship between Zn and glucose metabolism and lipid accumulation [89]. In mice with HDF-induced IR, supplementation with Zn chloride (20 mM ZnCl_2_) has been observed to reduce IR [90]. Zn meets with superoxide dismutase, and this enzyme is an antioxidant in the hepatocyte inflammation process. When Zn is depleted, its antioxidant power decreases, thus increasing lipid peroxidation and hepatocellular damage [91].

In Zn deficiency, various functions and regulatory mechanisms dependent on Zn absorption in the gastrointestinal tract are compromised. These mechanisms are also associated with MASLD development and progression, such as IR, hypertension, and dyslipidemia. However, the main mechanism affected by Zn deficiency is its antioxidant function, as it plays a central role in the adaptation of the endoplasmic reticulum to oxidative stress and inflammation attenuation, creating a vicious cycle in which Zn deficiency is caused by an increase in demand due to stress on the endoplasmic reticulum. In turn, this deficiency induces greater stress [92].

The effects of Zn gluconate supplementation have been studied in various liver diseases, specifically in MASLD. Razaei et al. [93] conducted a randomized clinical trial with 30 g of Zn gluconate supplementation vs. placebo in overweight patients with MASLD (both groups with dietary restriction and physical activity recommendations). The supplementation group showed significant decreases in AST (28.92 to 21.6 IU/L (*p* < 0.03)), total cholesterol (187.8 to 168.4 mg/dL (*p* < 0.003)), and low-density lipoprotein (107.9 to 85.40 mg/dL (*p* < 0.001)) levels after 8 weeks. However, no significant differences were observed in other indicators related to metabolism, oxidative stress, and inflammation.

### 3.5. Fatty Acids

Fatty acids are lipid molecules contained in fats, the macronutrient with the greatest energy content. They are components of various membranes and have different biological functions, such as cytokine production and the response they produce in tissues. Among the fatty acids derived from dietary lipids are polyunsaturated fatty acids (PUFAs) (ω-3 and ω-6); the dietary intake of these fatty acids is associated with the phospholipid composition of the immune cells’ membranes and the cells of the tissues where cytokines act [34].

Alpha-linolenic acid is an essential PUFA obtained from the diet, which is metabolized into ω-3 fatty acids, eicosapentaenoic acid (EPA) and docosahexaenoic acid (DHA). The main dietary sources of these fatty acids are flaxseed, canola, and soybean oils (alpha-linolenic acid) and fish such as salmon, tuna, sardines, cod, and hake (EPA and DHA) [94,95]. Therefore, the Western diet contains low amounts of ω-3 fatty acids by its nature [96].

ω-3 fatty acids have anti-inflammatory effects, improving T cell function. This effect is attributed to changes in membrane phospholipids, producing changes in cytokine- and lipid-derived inflammatory mediators and maintaining glutathione levels [34]. In MASLD, ω-3 fatty acids suppress de novo lipogenesis by suppressing the activity of the nuclear transcription factors peroxisome proliferator-activated receptor, sterol regulatory element-binding protein 1c, and carbohydrate-responsive element-binding protein [97,98].

Specifically, decreased levels of ω-3 fatty acids have been associated with reduced responses to increased cytokines and inflammation, so fatty acid supplementation has been associated with improved lymphocyte function and the promotion of the production of anti-inflammatory cytokines, such as IL-4 [99], and even more, with possible beneficial effects on the development of MASLD by promoting the balance of the intestinal microbiota, decreasing the increase in proinflammatory cytokines and oxidative stress, decreasing triglyceride synthesis, and attenuating inflammation by suppressing NFκB signaling [98].

Current American Heart Association guidelines [100] recommend ω-3 supplementation as an adjuvant in hypertriglyceridemia treatment; however, the effect on MASLD is still under study. A recent meta-analysis [101] of eight studies with a total of 6561 patients evaluated the beneficial effect of ω-3 supplementation in MASLD treatment, observing that supplementation significantly decreased the levels of AST (ESWMD = −3.73 IU/L, 95% CI: −5.93, −1.53, *p* < 0.001), ALT (ESWMD = −6.72 IU/L; 95% CI: −8.61, −4.84; *p* < 0.001), and γ-glutamyl transferase (ESWMD = −4.20 IU/L, 95% CI: −6.85, −1.55, *p* = 0.002). However, no differences in liver fat content were observed, highlighting that this result may be attributed to the heterogeneity of SLD measurement. Despite this possible effect, results from randomized clinical trials evaluating the effect of ω-3 supplementation at the histological level have not shown benefits [102,103].

### 3.6. Nucleotides

Nucleotides are essential compounds for various biological functions, including the response of immunoglobulins, the maintenance of intestinal cells, protein synthesis, and lipid metabolism. Although they are not essential compounds, they can be obtained from the diet through breast milk, meat, fish, beans, peas, lentils, and some types of mushrooms [104].

The main nucleotide associated with MASLD is NAD+, which is not directly obtained from the diet, but can be obtained through the consumption of its precursors, such as niacin, niacinamide, nicotinamide riboside (NR), and nicotinamide mononucleotide (NMN). Dietary sources include fish (such as salmon and tuna), lean meats, mushrooms, dairy products, whole grains, nuts, and seeds; however, the most readily available sources are found in dietary supplements containing NMN and NR [105].

NAD+ is a redox factor used by sirtuins; NAD+ is also a precursor of nicotinamide adenine dinucleotide phosphate. NAD+ has also been associated with decreased activation of HSC [105,106]. Redox homeostasis at the cellular level depends on the NAD+/NADH ratio; an imbalance in this ratio can lead to an excess of ROS production; therefore, supplementation with NAD+ or its precursors has been proposed as a regulator of NAD+/NADH levels [107].

The interaction between the endoplasmic reticulum and mitochondria through mitochondria-associated membranes plays an important role in the development of hepatic steatosis due to their function in pathways related to lipid synthesis, which, when altered, promote SLD [108]. Li et al. observed that NMN improved insulin resistance and HFD-induced hepatic steatosis in a murine model, enhancing cholesterol digestion and absorption due to improved mitochondrial dysfunction and endoplasmic reticulum oxidative stress, increasing hepatic NAD+ levels, which in turn increases mitochondria-associated membranes contact sites, mitigating lipid metabolic disturbances [109].

Regarding NR supplementation, various studies in murine models have shown that this supplementation increases NAD+ levels with effects in reducing triglycerides, fibrosis markers, and inflammation [105]. Recently, a randomized clinical trial with 111 patients with MASLD, Dellinger et al. [110] observed that supplementation with NR+ pterostilbene significantly decreased ALT and Gamma-glutamyl Transferase levels; however, no significant differences were observed in the primary endpoint, which was the reduction in liver fat.

One of the effects evaluated of nucleotides in MASLD is the possible reversal of liver damage through improved mitochondrial function; in a study with mice with HFD-induced hepatic steatosis and six human volunteers, it was observed that NAD+-boosting therapy could reverse steatosis by stimulating the exercise-related hormone fibronectin type III domain containing 5, also called irisin [111]. In this context, the effect of NAD+ precursors has also been evaluated; Mukherjee et al. [112] assessed the effect of supplementation with NR (500 mg/kg/day) in mice, observing that supplementation promoted liver regeneration through SIRT, whose substrate is NAD+, improving hepatic mitochondrial function and significantly decreasing hepatic triglyceride levels (*p* < 0.0001). Despite the promising results of nucleotide supplementation on mitochondrial function and other hepatic outcomes, it is important to consider that most evidence comes from animal models. Additionally, various factors such as age, gender, diet, and microbiota balance can affect the action of nucleotides [113].

Immunonutrients have different beneficial effects on MASLD pathways involving inflammation, oxidative stress, and lipid peroxidation, impacting clinical, metabolic, and hepatic outcomes. Therefore, immunonutrition could be an additional option for MASLD treatment; however, most of these positive effects have been observed in preclinical studies, with limited evidence from clinical trials or meta-analyses. Table 1 summarizes the available evidence regarding the effect of each immunonutrient in the MASLD scenario (Table 2).

## 4. Impact of Immunonutrients on MASLD Diet Pattern

The cornerstone of MASLD treatment is lifestyle modification, including physical activity and weight loss through dietary modifications. Regarding the latter, the effect of different dietary patterns on indicators of weight, inflammation, and liver function has been evaluated, including calorie restriction, the ketogenic diet, low-carbohydrate or low-lipid patterns, intermittent fasting, and the MedDiet [114]. The MedDiet has been shown to reduce intrahepatic lipid content, measured by magnetic resonance imaging (4–10%) and by 4.4% measured by biopsy [115]. Aller et al. [116] observed in 82 patients with hepatic steatosis, assessed by biopsy, that strong adherence to the MedDiet was higher in patients with a lower degree of steatosis and was associated with a lower risk of progression to steatohepatitis (0.43 (95% CI: 0.29–0.64)). Adherence to the MedDiet has also been associated with a reduced risk of liver fibrosis in patients with MASLD. In a study with 3325 patients with MASLD, Miryan et al. [117] determined the association of adherence to this dietary pattern with the presence of liver fibrosis, with significant results when the analysis was adjusted for sex, presence of diabetes mellitus, and physical activity (OR 0.666 (95% CI 0.474–0.937), *p* = 0.03), as well as when adjusted for weight and serum lipid blood levels (OR 0.656 (95% CI 0.466–0.923), *p* = 0.02). Currently, clinical practice guidelines from the major liver research associations (American Association for the Study of the Liver, European Association for the Study of the Liver, Asia-Pacific Association for the Study of the Liver, and Latin American Association for the Study of the Liver) recommend modifying the usual diet toward a MedDiet pattern in the treatment of MASLD [85,118,119,120].

The MedDiet is characterized by its high content of fruits, vegetables, whole grains, legumes, and minimally processed oilseeds, in addition to the inclusion of vegetable oils as the main source of fat. The protein intake of the MedDiet is based on the consumption of dairy products (mainly cheese and yogurt), poultry, and, to a lesser extent, red meat. This dietary pattern provides a large amount of health-beneficial compounds, the most important of which are polyphenols and polyunsaturated fatty acids such as ω-3. Due to these characteristics, the MedDiet is the dietary pattern with the greatest evidence of beneficial effects on health, mainly at the cardiovascular level, although its benefits have also been observed in metabolic diseases, neurological disorders, and some types of cancer [121,122,123].

Metabolic diseases are associated with an overproduction of ROS, which is associated with the activation of NF-KB, increasing inflammation levels [124]; in this scenario, due to its high content of polyphenols and fatty acids, the MedDiet has been associated with the reduction in inflammation. In a meta-analysis conducted by Koelman et al. [125] the MedDiet was shown to be the pattern with the best results in the reduction in inflammation biomarkers, such as IL-6 [MD –1.07 pg/mL (95% CI: –1.94, –0.20); I2: 96%], IL-1β [MD: –0.46 pg/mL (95% CI: –0.66, –0.25); I2: 0%], and C-reactive protein [MD: –1.00 mg/L (95% CI: –2.02, 0.01); I2: 100%], compared with other dietary patterns such as a low-fat diet, usual diet, or traditional diets. Adherence to the MedDiet has also been associated with a reduction in low-grade inflammation (B = −0.372, 95% CI −0.720 to −0.025) [126].

Due to the foods included in the MedDiet, the immunonutrient intake could contribute to anti-inflammatory and antioxidant effects. In a cross-sectional study of 685 patients, Bettermann et al. [127] observed that greater adherence to the MedDiet was positively associated with levels of immunonutrients (measured in plasma) such as glutathione (β = 0.02; 95% CI: 0.003, 0.03), and inversely associated with cysteine levels (β = –0.02; 95% CI: –0.04, –0.004), with an independent association between the level of adherence to the diet and a favorable thiol/disulfide redox status profile. Although these results come from a study with an apparently healthy population, we can assume that adherence to the MedDiet is directly related to the effects of immunonutrients derived from its characteristic foods.

The main immunonutrients contained in the characteristic foods of the MedDiet are ω-3 fatty acids, due to the contribution of vegetable fats, and polyphenols, due to the amount of fruits and vegetables included. The consumption of olive oil, the main source of lipids in the MedDiet, has been associated with the attenuation of adipose tissue hypertrophy and inflammation; specifically, the ω-3 content in olive oil improves antioxidant status in chronic diseases, such as MASLD [128]. Furthermore, the MedDiet is an important source of polyphenols, which, as previously mentioned, have a significant antioxidant effect. Specifically, polyphenols from the MedDiet have been shown to deregulate oxidative stress mediated by NF-κB and nicotinamide adenine dinucleotide phosphate [124].

Regarding the latter, it has been proposed to enrich the MedDiet pattern with polyphenols from green tea and to prefer the consumption of plant protein from plants with a high polyphenol content. This strategy, called the green-MedDiet, has been shown to have a greater impact on some cardiometabolic outcomes, such as the reduction in visceral fat. In a randomized clinical trial with 294 patients with at least one metabolic disorder, comparing the MedDiet with the green-MedDiet, Zelicha et al. [129] observed that patients randomized to the green-MedDiet group showed greater visceral fat loss compared to the MedDiet group (MedDiet: −6.0%, green-MedDiet: −14.1%; *p* < 0.05), regardless of sex, age, waist circumference, and weight loss. In addition to these results showing a beneficial effect of the green-MedDiet in patients with metabolic disorders, Yaskolka Meir A et al. evaluated the impact of this diet on intrahepatic fat loss assessed by magnetic resonance imaging, observing greater fat loss after 18 months of intervention with the green-MedDiet (−38.9%) compared to the MedDiet (−19.6%) (*p* = 0.03), after adjusting for weight loss [130]. The MedDiet with higher polyphenol content seems to be a strategy with beneficial effects on cardiovascular outcomes related to inflammation and liver fat content; however, despite these important results, clinical trials have not evaluated the effect of this diet in patients with MASLD.

While immunonutrient supplementation could be part of the treatment of MASLD, following a MedDiet eating pattern, which includes immunonutrients, is a more available and cost-effective strategy. However, despite the MedDiet’s obvious benefits, acceptability and adherence depend not only on clinical factors but also on patients’ socioeconomic status, primarily in geographic regions where the availability of the MedDiet’s signature foods is low, which significantly increases its cost [131].

In order to address these MedDiet limitations, another regional dietary pattern has been proposed: the Milpa diet, based on the consumption of foods from the milpa system, which corresponds to the agricultural production system characteristic of Mesoamerica, where foods such as corn, legumes (beans), squash, chili peppers, and tomatoes are obtained through polyculture [132]. The consumption of fats from avocado, animal proteins such as eggs and cottage cheese (dairy), other cereals such as amaranth, vegetables such as nopales, garlic and onion, fruits such as prickly pears, papaya and guava, and oilseeds such as chia, peanuts and pumpkin seeds constitutes the pattern of the Milpa diet [133].

The contribution of beneficial components for health in the Milpa diet has been compared to that of the MedDiet, due to the inclusion of foods, mainly of plant origin, with a high content of antioxidants, polyphenols, and vitamins A, C and E [134]. The Milpa diet also represents the contribution of a significant amount of immunonutrients associated with beneficial effects in MASLD: (1) amino acids from beans, garlic and onion, (2) polyphenols from corn (catechins), beans, amaranth, and chia, (3) vitamins characteristic of fruits, vegetables and corn (vitamin C) and vegetable oils (vitamin E), (4) micronutrients contained in seeds such as peanuts, pumpkin, and corn (Zn), and (5) n-3 fatty acids contained in pumpkin seeds, peanuts, chia, and avocado [133,135]. Although the effect of the Milpa diet requires further evidence, it has been proposed that, due to its content of compounds that have been shown to have beneficial effects on various metabolic scenarios, it could be a strategy to be implemented in patients with MASLD, mainly in regions with a higher prevalence of the disease due to genetic factors, where adherence to the MedDiet depends on availability, and where the Milpa diet represents an accessible option concordant with traditional diets.

One of the key factors in managing MASLD is the regulation of gut microbiota. In recent years, evidence demonstrating the role of microbiota in the pathophysiology of MASLD has been increasing; the gut–liver axis interacts with various signaling and inflammatory pathways, stemming from microbiota function in metabolizing fiber, bile acids, and nutrients, and generating metabolites that may have an effect in mitigating MASLD [136,137].

In MASLD patients, dysbiosis has been linked to the severity of liver steatosis. These patients show an increased abundance of Enterobacteriaceae, particularly from the *Pseudomonadota phylum*, which is associated with inflammation. Conversely, *Ruminococcaceae* and *Rikenellaceae*, bacterial families belonging to the *Firmicutes* and *Bacteroidetes* phyla, respectively, involved in fiber fermentation and short-chain fatty acid production, are decreased in these patients [138].

Due to microbiota-diet interactions, it has been proposed that dietary pattern modification could have a positive effect on microbiota composition. In this context, the MedDiet has been shown to significantly reduce the species of *Ruminococcus* characterized by their association with inflammation (*R. gnavus* and *R. torques*) [136,139]. Modification of microbiota composition through the MedDiet is attributable to the consumption of dietary fiber, prebiotics, and immunonutrients, such as ω-3 fatty acids, which increase the number of species that synthesize short-chain fatty acids, including *Clostridium leptum* and *Eubacterium rectale* [140].

The most robust evidence of the beneficial effect of immunonutrients on the gut microbiota is attributed to polyphenols, which therapeutic effects related to the improvement of lipid profiles, systemic inflammation, and scavenging of reactive oxygen species are attributed to their close relationship with the gut microbiome [59]. The green-MedDiet has also been shown to have a significant impact on microbiota composition. Rinott et al. [141] evaluated the effect of the green-MedDiet on the microbiota composition of patients with obesity and dyslipidemia, randomized into three groups (healthy dietary guidelines, MedDiet, and green-MedDiet). After six months of intervention, the green-MedDiet showed enrichment of *Prevotella*, a bacterium belonging to the *Bacteroidetes* phylum, which has been associated with improved glucose metabolism and effects on weight loss. Specifically in patients with MASLD, curcumin supplementation has been shown to increase the amount of *Bacteroidetes* significantly [73].

Even the observed beneficial changes in microbiota due to diet modifications are influenced by many factors that affect microbiome composition. These factors are not limited to the presence of hepatic disease but also include genetic predisposition, drug exposure, dietary history, geographic location, and birth factors. All of these serve as significant limitations when generalizing the study results.

## 5. Future Perspectives

In chronic liver diseases (such as cirrhosis), immunonutrition has demonstrated a beneficial effect due to the interaction of immunonutrients in inflammation pathways and oxidative stress. In this way, this nutritional strategy could play an important role in the multidisciplinary treatment MASLD field. So far, the evaluation of the effects of immunonutrients in MASLD has been based on the reduction in inflammatory markers and liver function tests at the systemic level, which are considered good response markers. However, over time and with the significant increase in prevalence, the endpoints for MASLD have become more stringent, creating a scenario where normalization of liver enzymes is no longer sufficient. Therefore, it is necessary to design clinical trials with more rigorous outcomes that evaluate the effect of immunonutrients in MASLD. It seems only a matter of time before this nutritional strategy is considered an adjuvant option for MASLD treatment, either through modification of dietary patterns or specific immunonutrient supplementation.

Current evidence and clinical practice guidelines for the treatment of MASLD show that dietary modification is a fundamental factor in the management of this liver disease. Changing traditional Western diets or those with a high consumption of ultra-processed foods toward healthy dietary patterns has been shown to have beneficial effects on various MASLD indicators; however, from a pathophysiological point of view, the MedDiet pattern has been shown to have a greater impact on cardiovascular and metabolic indicators, primarily due to the presence of chemical components with beneficial effects on health and the provision of immunonutrients; hence its close beneficial relationship with MASLD.

Nonetheless, the main limitation of changing traditional dietary patterns is medium- and long-term adherence, especially when they are aimed at treating rather than preventing the disease. With this in mind, the role of diet may not be as relevant in the early stages of the currently available pharmacological treatments for MASLD, but the importance of diet will likely increase in a fundamental aspect: maintaining the results achieved with pharmacological treatment. To date, it has only been evaluated over 52-week periods. To maintain these effects in the long term, pharmacological treatment must be accompanied by lifestyle changes regarding diet and physical activity indefinitely.

On the other hand, the costs of the MedDiet may have economic implications that limit long-term adherence, especially in regions with less availability of the foods that are characteristic of this dietary pattern. As for the already established benefits of MedDiet components in patients with MASLD, including immunonutrients, dietary modification with sociodemographic approaches and adjustments allows for the enrichment of polyphenol intake and the addition of foods containing other immunonutrients associated with beneficial effects in MASLD, such as amino acids, vitamins C and E, Zn, and nucleotides.

## 6. Conclusions

Immunonutrition is a strategy that has so far demonstrated beneficial effects in MASLD; however, evidence still needs to be expanded on the effect of specific immunonutrient supplementation on hepatic, metabolic, and cardiovascular outcomes in these patients. Future development of clinical trials should also evaluate the availability, cost, and access to foods containing immunonutrients or specific supplements, according to the clinical characteristics and geographic region of the population.

## Figures and Tables

**Table 1:** Dietary sources of immunonutrients.

Immunonutrient	Foods
Cysteine	Meat, egg, and whole grains
Arginine	Meat, fish, nuts, wheat germ, whole grains, soy, and dairy products
Glutamine	Meat, eggs, fish, dairy products, wheat, tofu, and fermented products
BCAA	Meat, fish, dairy, eggs
Polyphenols	Vegetables, fruits, cocoa, legumes, curcuma, green tea, corn, beans, amaranth, chia
Vitamin C	Citrus fruits, berries, tomatoes, peppers, corn, leafy greens, broccoli, kale, and parsley
Vitamin E	Vegetable oils (wheat germ, sunflower, corn, soybean), nuts, almonds, peanuts, hazelnuts, green leafy vegetables, broccoli and spinach, kiwi, mango, and fortified cereals
Zinc	Seafood, beef, pork, chicken, cheese, almonds, sunflowers, pumpkin seeds, and cashews
ω-3 fatty acids	Flaxseed, canola, and soybean oils, pumpkin seeds, peanuts, chia, avocado, salmon, tuna, sardines, cod, and hake
Cysteine	Meat, egg, and whole grains
Nucleotides	Meat, fish, beans, peas, lentil, mushroom

**Table 2:** Effects of immunonutrients on MASLD.

Type	Immunonutrient	Year Author Type of Study	Population	Intervention	Principal Findings
Amino acids	Cysteine	2021 Hang W et al. [32] Basic	HFD induced MASLD mice	NAC vs. PBS	Decrease hepatic TG accumulation Preserved mitochondrial function Preventing ROS production
		2023 Tran M et al. [30] Basic	SerpinAN3 knock-out MASLD induced mice	Induced SerpinAN3	Decrease in leptin and insulin levels Improved glucose tolerance
	Arginine	2015 Abu-Serie MM et al. [40] Basic	Induced steatohepatitis murine model	500 mg/kg Arginine	Decrease levels of lipid peroxidation and TNF-α Decrease the activity of CYP2E1 Increase the activity of nitric oxide and endothelial nitric oxide
	Glutamine	2014 Lin Z et al. [49] Basic	HFD-induced MASLD rats	1 g/kg/day Glutamine	Decrease in oxidative stress, TNF-α, and MDA
	BCAA	2016 Honda T et al. [54] Basic	CDHFD MASLD murine model	0.23 g isoleucine/g + 0.46 g leucine/g + 0.28 g valine/g	Decrease in serum ALT and glucose Decrease in hepatic TG Decrease fatty acid synthetase activity
Polyphenols	EGCG	2021 Wu D et al. [69] Basic	Liver cell line and HFD-induced MASLD murine model	50 mg/kg/day EGCG	Decrease mitochondrial-dependent apoptosis Increase autophagy
		2021 Du Y et al. [70] Basic	Fructose + HFD-induced MASLD murine model	EGCG low dose (25 mg/kg/day) vs. high dose (50 mg/kg/day)	Decrease in serum cholesterol Decrease hepatic TG Decrease in steatosis and steatohepatitis scores
	Curcumin	2023 Wu J et al. [76] Basic	HFD-induced MASLD murine model	Curcumin supplement	Improvement of hepatic endothelial function Decrease hepatic lipid accumulation and inflammation
		2023 Lukkunaprasit T et al. [66] Meta-analysis	16 RCTs in MASLD patients	Curcumin supplementation	Decrease in BMI Improvement of AST, ALT Decrease fasting glucose and total cholesterol Steatosis resolution Decrease steatosis severity
		2024 He Y et al. [73] RCT	80 MASLD patients	500 mg/day Curcumin vs. placebo	Decrease hepatic steatosis (dB/m) Decrease in BMI, free fatty acids, TG, fasting glucose, and HbA1c
Vitamins	Vitamin C	2021 He Z et al. [79] RCT	84 MASLD patients	1000 mg/day of Vitamin C	Decrease AST and ALT levels Improvement of HOMA-IR
		2022 Lee S et al. [78] Basic	HFD-induced MASLD mice	Megadose of Vitamin C	Less lobular inflammation and fat accumulation Decrease TG and free fatty acids accumulation
	Vitamin E	2010 Sanyal AJ et al. [84] RCT—PIVENS study	247 NASH patients	800 IU/day of Vitamin E	Decrease ALT and AST levels Decrease in lobular inflammation and hepatocellular ballooning Decrease in NAS Score
		2024 Lahmi A et al. [86] Basic	HFD-induced MASLD murine model	200 mg/kg/day of Vitamin E and 200 mg/kg/day of Vitamin E + 50 mg/kg/day of thymol	Decrease in AST and ALT levels Cytoarchitectural changes in hepatocytes
Trace element	Zinc	2023 Razei SMA et al. [93] RCT	50 MASLD patients	30 mg/day Zinc Gluconate vs. placebo	Decrease in AST, LDL, and total cholesterol levels
Fatty acids	ω-3	2023 Musazadeh V et al. [101] Meta-analysis	6561 MASLD patients	ω-3 supplementation	Decrease in AST, ALT, and GGT levels
Nucleotides	NAD+	2021 Li D et al. [111] Basic	NAFLD murine model	NAD+-Boosting supplementation	Reversion of liver steatosis
	NR	2021 Mukherjee et al. [112] Basic	Hepatocyte-specific SIRT1-ko mice	500 mg/day of NR	Hepatic regeneration Improve mitochondrial function Decrease hepatic TG
	NMN	2023 Dellinger, RW et al. [110] RCT	111 MASLD patients	NR+Pterostilbene supplementation	Decrease in AST and GGT serum levels
		2024 Li Y et al. [109] Basic	HFD SLD induced murine model	500 mg/L od NMN on pure water	Improve IR and mitochondrial dysfunction

HFD High-Fat Diet; MASLD Metabolic Dysfunction-Associated Steatotic Liver Disease; NAC N-acetyl cysteine; PBS Phosphate-Buffered Saline; TG Triglycerides; ROS Reactive Oxygen Species; PPAR-α Peroxisome Proliferator-Activated Receptor alpha; TNF-α Tumor Necrosis Factor alpha; CYP2E1 Cytochrome P450 2E1; MDA Malondialdehyde; CD Choline Deficient; ALT Alanine Transaminase; EGCG Epigallocatechin gallate; RCT Randomized Clinical Trial; BMI Body Mass Index; AST Aspartate aminotransferase; HbA1c Glycated Hemoglobin; HOMA-IR Homeostasis Model Assessment of Insulin Resistance; MASH Metabolic dysfunction-Associated Steatohepatitis; NAS NAFLD Activity Score; ALP Alkaline Phosphatase, LDL Low-Density Lipoproteins, GGT Gamma-glutamyl Transferase; NAD Nicotinamide Adenine Dinucleotide; NR Nicotinamide Riboside; SIRT1 Sirtuin 1; NMN Nicotinamide Mononucleotide; SLD Steatotic Liver Disease.

## Data Availability

Not applicable.

## Abbreviations

The following abbreviations are used in this manuscript:

MASLD	Metabolic Dysfunction-Associated Steatotic Liver Disease
MASH	Metabolic Dysfunction-Associated Steatohepatitis
NF-κB	Nuclear Factor kappa B
IR	Insulin Resistance
ω-3	Omega-3 Fatty Acid
ROS	Reactive Oxygen Species
HSC	Hepatic Stellate Cells
AMPK	AMP activated protein kinase
mTOR	Mechanistic Target of Rapamycin
SIRT1	Sirtuin 1
SREBP1c	Sterol Regulatory Element-Binding Protein
Zn	Zinc
SPARC	Secretory Acidic Protein Rich in Cysteine
SLD	Steatotic Liver Disease
NAC	N-acetylcysteine
AST	Aspartate aminotransferase
ALT	Alanine aminotransferase
ALP	Alkaline phosphatase
TNF-α	Tumor Necrosis Factor-alpha
HFD	High-Fat Diet
BCAA	Branched Chain Amino acids
MedDiet	Mediterranean Diet
NAD	Nicotinamide Adenine Dinucleotide
EGCG	Epigallocatechin-3-gallate
PUFA	Polyunsaturated Fatty Acids
NR	Nicotinamide Riboside
NMN	Nicotinamide Mononucleotide
